# Features of Non-Alcoholic Beer on Cardiovascular Biomarkers. Can It Be a Substitute for Conventional Beer?

**DOI:** 10.3390/nu15010173

**Published:** 2022-12-30

**Authors:** Marco Sancén, Asier Léniz, María Teresa Macarulla, Marcela González, Iñaki Milton-Laskibar, María P. Portillo

**Affiliations:** 1 Nutrition and Obesity Group, Department of Nutrition and Food Science, Faculty of Pharmacy and Lucio Lascaray Research Center, University of the Basque Country (UPV/EHU), 01006 Vitoria-Gasteiz, Spain; 2 Vitoria-Gasteiz Nursing School, Osakidetza-Basque Health Service-University of the Basque Country (UPV/EHU), 01009 Vitoria-Gasteiz, Spain; 3 BIOARABA Institute of Health, 01006 Vitoria-Gasteiz, Spain; 4 CIBERobn Physiopathology of Obesity and Nutrition, Institute of Health Carlos III, 01006 Vitoria, Spain; 5 Nutrition and Food Science Department, Faculty of Biochemistry and Biological Sciences, National University of Litoral and National Scientific and Technical Research Council (CONICET), Santa Fe 3000, Argentina

**Keywords:** beer, non-alcoholic beer, cardiovascular markers, (poly)phenols, alcohol

## Abstract

Numerous studies have revealed the beneficial effects of moderate beer consumption on cardiovascular diseases. However, the presence of alcohol in beer can represent a matter of concern, since alcohol intake poses a risk to some individuals. Additionally, adults who are life-long abstainers should not be encouraged to consume alcohol for health purposes. Consequently, the benefits of beer consumption remain a controversial issue. In this scenario, the present review gathers the reported information concerning the cardiovascular effects of non-alcoholic beer, and makes a comparison between these effects and those of conventional beer. Despite the scarcity of published results to date describing the effects of non-alcoholic beer consumption, the available literature indicates that it is more effective than conventional beer in preventing oxidative stress (lower lipid and protein oxidation), preserving the endothelial function (lower endothelial dysfunction) and inhibiting thrombogenic activity (lowered oxidized LDL). By contrast, conventional beer has shown to induce greater increases in HDL-cholesterol levels (known as a cardiovascular protective factor) compared to non-alcoholic beer. This effect cannot be solely attributed to alcohol content, since the polyphenol content in conventional beer tends to be higher than that found in non-alcoholic beer.

## 1. Introduction

Beer is the most widely consumed alcoholic beverage throughout the world. Although its main component is water, it also contains nutrients, such as carbohydrates, amino acids, minerals (mainly fluoride and silicon), vitamins (the quantity of folate and choline being relatively significant) and bioactive compounds such as polyphenols [1,2]. Polyphenols come mainly from the hop, and are used as a bittering and flavoring agent in the elaboration process [3]. Alcohol content in regular beer varies between 3.5% and 10% by volume [2].

Numerous studies have demonstrated the beneficial effects of moderate beer consumption on cardiovascular diseases [4]. These pathologies raise great concern because, according to the World Health Organization, they also represent the majority of deaths from chronic diseases; that is, 17.9 million people a year [5]. In the recent review reported by Marcos et al. (2021), the authors concluded that moderate beer drinking decreased cardiovascular risk and overall mortality [6]. They defined moderate consumption as the intake of 10–16 g alcohol/day (1 beer/day) for women and 20–28 g alcohol/day (1–2 beers/day) for men, providing that the consumption was distributed throughout the week, with no heavy episodic or “binge drinking” on a single occasion, especially during weekends. Some of the positive effects of beer on cardiovascular disease markers are an increase in high-density lipoprotein cholesterol (HDL), a reduction in arterial stiffness and a decrease in fibrinogen, platelet activation and aggregation, oxidative stress and inflammatory parameters [7].

In spite of these beneficial effects, the presence of alcohol in beer can represent a matter of concern for those advised against alcohol consumption (pregnant women, people suffering from certain diseases, people treated with specific drugs, etc.) and thus unable to benefit from the beverage’s positive effects. On the other hand, adults who are life-long abstainers should not be encouraged to consume alcohol based on health reasons. Moreover, governments are launching national campaigns aimed at reducing alcohol intake among the population, because it has been reported that there is no safe intake of this component [8]. Consequently, the benefits of drinking beer remain a controversial issue [9].

In this scenario, the present review is aimed at gathering the reported information concerning the cardiovascular effects of non-alcoholic beer, as well as comparing these effects with those of conventional beer. 

## 2. Studies Addressing the Beneficial Effects of Non-Alcoholic Beer

A number of studies have analyzed the effects of non-alcoholic beer consumption, but have not compared them with those produced by conventional beer. Two of these studies were carried out in nuns, a very interesting population because, due to their lifestyle, it is more homogeneous than other population groups (Table 1).

The study published by Martinez-Alvarez et al. (2009) was carried out in 29 postmenopausal nuns (a population particularly at risk for atherosclerotic disease), aged 58–73 years [10]. They were given two 250 mL/day non-alcoholic lager beers with normal daily meals, for 45 days. At the end of the study, total cholesterol, HDL-cholesterol, LDL-cholesterol and triglycerides did not change from baseline values, except in subjects with cholesterol levels above 240 mg/dL, who showed lower levels after supplementation. These results implies that the non-alcoholic beer only acted when hypercholesterolemia was present. Moreover, circulating oxidized LDL-cholesterol levels decreased significantly, suggesting a weaker susceptibility to LDL-cholesterol oxidation. Looking further into oxidative metabolism, thiobarbiturate acid-reactive substances (TBARS) and carbonyl group concentrations were significantly reduced at the end of the supplementation period, compared to baseline values. Thus, lipid peroxidation and protein oxidation decreased after dietary supplementation with non-alcoholic beer. In addition, a significant boost in plasma α-tocopherol concentration (with well-known antioxidant activity) was also observed, as well as an increase in erythrocyte glutathione content. Regarding markers related to inflammation, the fact that the circulating concentrations of serum C-reactive protein (CRP), C3 and C4 complements, IL-1, IL-6 and TNFα were unaffected suggests that non-alcoholic beer consumption did not alter inflammatory defense capacity. Thus, the authors concluded that non-alcoholic beer consumption induced an antioxidant effect, which represents protection from cardiovascular diseases. It was further suggested that this effect may be due to the polyphenol content of beer, which are mainly flavonoids and melanoidins.

Following a six-month period, participants in this study were administered 400 mg/d of commercial hop (Elusan1), which is one of the main contributors to the polyphenol content of beer, for 30 days. A significant decrease in triglyceride and total cholesterol concentrations was observed, along with a reduction in oxidized LDL. In addition, TBARS and carbonyl group content was significantly lowered and a further increase in reduced glutathione (GSH) and α-tocopherol were also observed. With regard to inflammation, hop supplementation led to a significant decrease in Complement C3 fraction, CRP and IL-6 levels, indicating that this second intervention did have an anti-inflammatory effect [11]. Although not through non-alcoholic beer, the fact that hop administration resulted in decreased inflammatory parameters can be due to its greater number of polyphenols. 

In the study reported by Scherr et al. (2012) the effects of non-alcoholic beer administration were compared with those of a placebo containing the same ingredients as non-alcoholic beer, except for polyphenols [12]. In this study 277 healthy German male runners aged 20–60 years were randomly assigned to drink 1–1.5 L/d of non-alcoholic beer or placebo for three weeks before the Munich Marathon, during the race and two weeks after. The results showed that, compared with the placebo, the inflammation parameters measured (serum interleukin-6 and blood leukocyte counts) were significantly reduced by non-alcoholic beer ingestion. In addition, the incidence of Upper Respiratory Tract Illness (URTI) caused by transient immune dysfunction resulting from strenuous exercise after the race was significantly higher in the placebo group, compared to the non-alcoholic beer group. The authors concluded that the consumption of non-alcoholic beer containing polyphenols for three weeks before the marathon reduced inflammation after the race. Moreover, extended intake of non-alcoholic beer for two weeks after the race reduced the incidence of clinically relevant respiratory infections. Since the difference between non-alcoholic beer and placebo relies on its polyphenol content, it can be proposed that the positive effects observed after ingestion of non-alcoholic beers are due to these compounds.

More recently, Macías-Rodríguez et al. (2020) reported a study carried out in 43 Mexican individuals aged between 18 and 70 years (21 controls and 22 non-alcoholic beer drinkers), of whom 26 were women and 17 men [13]. All of them had non-alcoholic liver cirrhosis and they had all undergone an intervention which combined a dietary component and physical exercise. The control group received 300 mL of water, while the intervention group ingested 300 mL (one can) of non-alcoholic beer, for a period of eight weeks. Among subjects with endothelial dysfunction at baseline, 72.7% in the control group showed an improvement by the end of the study; the percentage of individuals who experienced this improvement in the intervention group was of 83.3%. This change was significant in both groups and significantly greater in the intervention group than in the control group. Although there was improvement in endothelial function as a result of exercise in both groups, non-alcoholic beer consumption provided additional benefits.

## 3. Studies Addressing Comparative Effects of Conventional Beer and Non-Alcoholic Beer

The effects of non-alcoholic beer have been compared with those of conventional beer in a number of studies. These interesting pieces of research shed light on the open question of whether the beneficial effects of fermented alcoholic beverages (mainly) depend on their alcohol content or on their non-alcoholic components (Table 2).

The work reported by Bassus et al. (2004) included 12 healthy men, aged 19–36 years, who sequentially consumed three liters of conventional beer (4 *v*/*v*% ethanol), non-alcoholic beer or 4% ethanol/water (*v*/*v*) at a 3-hour interval (about one liter/hour), with 4-week rest periods between each intervention [14]. Non-alcoholic beer consumption significantly reduced the expression of activated fibrinogen receptor (PAC-1), a glycoprotein complex that is converted to fibrin during vascular injury by thrombin and then to a fibrin-based blood clot, along with the platelet activation marker CD62, by 15% and 25% from baseline, respectively. In the case of 4% ethanol, there was a decrease in the expression of both platelet activation markers, which did not reach statistical significance. Conventional beer had no effect on CD62 and PAC-1 expression, indicating that the effects of ethanol and non-alcoholic components on PAC-1 and CD62 are antagonistic.

Additionally, whereas non-alcoholic beer consumption induced a slight decrease in clotting factor VII (FVIIc) activity that did not reach statistical significance, ethanol or alcoholic beer consumption resulted in a moderate increase in this parameter, with the change being statistically significant 3.5 h after consumption. On the other hand, a significant reduction in endogenous thrombin potential (ETP), which represents the balance between pro- and anti-coagulant forces operating in plasma and is utilized to investigate hyper- and hypo-coagulability, was observed for non-alcoholic beer 1.5 h and 3.5 h after consumption. By contrast, conventional beer or ethanol consumption resulted in a significant increase in ETP and thus activation of coagulation. None of the beverages had a significant effect on prothrombin time, activated partial thromboplastin time or fibrinogen level. Regarding monocyte platelet aggregates (MPA), a sepsis prognostic indicator, consumption of three liters of 4% ethanol or conventional beer reduced this parameter by 10–20%, whereas consumption of three liters of non-alcoholic beer reduced it by up to 40%. Thus, beer and ethanol appear to have procoagulatory effects, whereas the non-alcoholic components have no impact on this activity.

The results of this study therefore show that both conventional and non-alcoholic beer have anti-platelet effects, although coagulation is inhibited by non-alcoholic beer and stimulated by beer and ethanol. Thus, non-alcoholic beer’s effects on platelets and coagulation seem to indicate that its consumption may be beneficial in ischemic heart disease.

Imhof et al. (2008) carried out a study in 49 healthy, non-smoker German men and women, aged between 22 and 56 years [15]. Participants were divided into six experimental groups and, after a washout period of at least two weeks, they were administered ethanol (at a concentration of 12.5%), beer (5.6% alcohol) and red wine (12.5% alcohol) in amounts that provided 30 g of ethanol per day (g/d) for men and 20 g/d for women, or the same amount of dealcoholized beer or dealcoholized red wine (of the same brand) or water (control group) for three weeks. Interestingly, in this study both types of beer showed similar amounts of polyphenols. In the present review only the comparison between both types of beer has been analyzed. Beer consumption did not modify HDL-c. When the migration of monocytes was measured ex vivo, using a modified Boyden chamber, neither conventional beer nor non-alcoholic beer affected this parameter significantly. Similarly, other parameters of inflammation, such as intercellular adhesion molecule-1 (ICAM) and TNF α, remained unchanged after beer consumption. 

The same group further addressed a study devoted to analyzing the effects on plasma adiponectin in a cohort showing similar characteristics to the previous study (72 participants, without specifying the distribution between sexes) [16]. The authors observed a significant increase in this adipokine after ingestion of conventional beer in men. By contrast, non-alcoholic beer did not have a substantial effect. These results are in line with those reported in other studies in which adiponectin levels were also measured and which have been previously described in the present review [17,19].

Beulens et al. (2008a) carried out a cross-cohort study in 20 non-smoker Dutch male subjects, aged 18–25 years [17]. Participants were classified into normal weight and overweight subjects and received three cans (990 mL) of conventional or non-alcoholic beer for three weeks, with a washout period of one week. After each experimental period, an oral glucose tolerance test (OGTT) was performed, where peripheral blood was extracted. Compared with non-alcoholic beer, the intake of conventional beer produced an increase in adiponectin and ghrelin concentrations, as well as a decrease in acylation stimulating protein (ASP) levels. Despite what might be expected, due to the known positive effects of adiponectin on glycemic control, the change observed in this adipokine did not lead to an insulin sensitivity amelioration in the group that consumed conventional beer. In fact, only small changes were noticed in the OGTT, such as a decrease in glucose concentration after two hours. In light of these results, the authors hypothesized that this change might precede an increase in insulin sensitivity, but unfortunately this could not be tested due to the short duration of the study. Because these findings were not evidenced with alcohol-free beer, the change in adiponectin levels being related to its phenolic compounds seems implausible, although not to its ethanol content.

In another research study addressing the same cohort, serum lipids and lipoprotein-associated phospholipase A2 (Lp-PLA2) as well as inflammation and oxidative stress markers were measured [18]. Conventional beer consumption led to an increase in HDL-cholesterol and a decrease in LDL-cholesterol when compared with non-alcoholic beer consumption. No differences were observed in Lp-PLA2 activity, C-reactive protein or systolic blood pressure. Diastolic blood pressure tended to increase after conventional beer intake, but this result was not statistically significant. In the same way, urinary F2-isoprostanes tended to increment after consumption of conventional beer compared to ingestion of non-alcoholic beer. In addition, liver enzymes gamma-glutamyl transferase (GGT) and aspartate transaminase (AST) were slightly elevated following conventional beer intake. In summary, it can be stated that neither the consumption of conventional beer nor that of non-alcoholic beer modified the activity of Lp-PLA2. However, whereas conventional beer, due to its alcoholic component, increased oxidative stress in all subjects and liver enzyme levels in overweight subjects, the alcohol-free beer did not induce these negative effects. Thus, there was a less favorable response to conventional beer consumption despite its positive effects on cholesterol levels.

In the study reported by Joosten et al. (2011), adiponectin concentration was analyzed in non-smoker women aged 20–40 years and treated with oral contraceptives [19]. Participants were randomly assigned to two groups, according to the type of beer (both Amstel, The Netherlands): one group received two cans of conventional beer (660 mL ∼26 g alcohol) and the other received two cans of non-alcoholic beer (0.2 g alcohol). The beverages were administered daily for three weeks, followed by a 1-week washout and finally a 3-week new intervention period with the other beer.

In line with the results found by Beulens et al. (2008a), among male subjects of similar age, plasma adiponectin concentrations were higher after moderate consumption of conventional beer compared to non-alcoholic beer, but there were no differences in serum glucose, insulin, hemoglobin A1c or triglyceride levels between the two intervention groups [17]. Thus, changes in adiponectin levels did not lead to an improvement in glycemic control. 

In the study reported by Karatzi et al. (2013), 17 healthy, non-smoker Greek men, with a mean age of 28.5 ± 5.2 years, were divided into two experimental groups who consumed on different occasions, with at least a one-week interval between them, any of the following combinations. In the first group, participants received 400 mL of beer and 400 mL of water; the second group received 800 mL of non-alcoholic beer (same amount of polyphenols as conventional beer, 48 mg) [20]. Aortic stiffness was significantly and similarly reduced with both types of beer. However, endothelial function was only significantly improved after consumption of conventional beer. Although wave reflexes, a marker reflecting the augmentation of blood pressure due to returning reflected waves from distal circulation sites, were significantly reduced in both interventions, the decrease was greater following conventional beer consumption than following non-alcoholic beer intake. Pulse pressure amplification (brachial/aortic), associated with both arterial stiffness and wave reflections as well as with heart rate and the classical cardiovascular risk factors, increased with both beers. Therefore, the work shows that conventional beer consumption acutely improved arterial properties in apparently healthy men, which could be related to the synergistic effects of the alcohol content and the antioxidants present in these beverages.

In a study carried out by Chiva-Blanch et al. (2014), the effects of conventional and non-alcoholic beer on the number of stem cells derived from bone marrow were compared [21]. This parameter is very important in the repair and maintenance of endothelial integrity and function and is considered a surrogate marker of vascular function and cumulative cardiovascular risk. The study was carried out in 33 men, aged between 55 and 75 years, with high cardiovascular risk (type 2 diabetes, high blood pressure, dyslipidemia, overweight or obese, smoking and/or a family history of cardiovascular disease). They were administered 660 mL of lager beer (30 g alcohol/day and 1209 mg total polyphenols) or 990 mL of non-alcoholic lager beer (<1 g of ethanol and 1243 mg of total polyphenols). Participants were randomized in a crossover design in intervention sequences of four weeks each, in which the studied beverages were provided. Following the conventional or non-alcoholic beer interventions, the number of circulating endothelial progenitor cells increased significantly. Serum-soluble stromal cell-derived factor 1 increased significantly after beer ingestion. The authors concluded that, in the population at high cardiovascular risk, the mechanism that can explain the cardioprotective effects of beer was related to the increase in the number of circulating endothelial progenitor cells in peripheral blood caused by the non-alcoholic fraction of beer.

In the same cohort, and using the same experimental design, the researchers conducted another study focused on the effect of ethanol and beer polyphenols on biomarkers of atherosclerosis [22]. They found that systolic blood pressure decreased after the intervention with non-alcoholic beer, while no difference was observed after the ingestion of conventional beer. However, following the last interventions, HDL cholesterol, ApoA-I, ApoA-II and adiponectin increased from baseline and also when compared to the non-alcoholic beer intervention. Homocysteine concentration decreased and serum folic acid was boosted, although only after intervention with non-alcoholic beer. Serum fibrinogen was reduced following conventional beer intake, but not with non-alcoholic beer intervention. No differences were observed in the rest of the coagulation parameters. In addition, serum concentrations of soluble inflammation biomarkers and leukocyte adhesion decreased after the intervention with alcoholic and non-alcoholic beer. In conclusion, the absorption of polyphenols associated to conventional and non-alcoholic beer consumption could be involved in the protective effects on the cardiovascular system observed by the authors.

The work reported by Padro et al. (2018) included 21 men and 15 women aged 40–60 years, non-smokers, overweight or grade 1 obese with healthy conditions, i.e., without other cardiovascular risk factors (dyslipidemia, type 2 diabetes or hypertension) [23]. Participants received conventional lager beer (15 g ethanol and 604 mg polyphenols/can) or non-alcoholic lager beer (0.0 g alcohol and 414 mg polyphenols/can). The men consumed two cans/day (660 mL of beer, i.e., 30 g of alcohol) and the women one can/day (330 mL of beer, i.e., 15 g of alcohol). After a 4-week adaptation period the intervention was carried out for four additional weeks with beer (depending on the assigned group), followed by a 4-week washout, and finally a 4-week new intervention period with the other beer. Serum concentrations of total cholesterol, HDL-cholesterol, LDL-cholesterol and triglycerides, as well as systemic inflammatory markers such as CRP, IL6 and TNF-α, showed no significant changes with either conventional or non-alcoholic beer consumption. Moreover, moderate beer intake (conventional or non-alcoholic) had no detrimental vascular effects (Framingham Risk Score did not increase). On the contrary, beer consumption was associated with favorable effects, such as an improvement of the atheroprotective properties of HDL, increasing its ability to protect from LDL oxidation (both beers) and boosting cholesterol efflux from macrophages (only conventional beer), which may prevent lipid deposition in vessel wall. These results demonstrate beer’s antioxidant effect. The researchers did not provide any explanation for the differential effects of the two beers. 

Muñoz-Garcia et al. (2021) investigated whether regular and moderate beer consumption modulated the functional behavior of human macrophages when exposed to external pro-inflammatory stimulus [24]. The study included 36 healthy regular beer drinkers (21 men and 15 women) aged 40–60 years, non-smokers, overweight or featuring grade 1 obese. They were given non-alcoholic beer (0.0 g alcohol and 414 mg polyphenols/can) or conventional beer with 5.7% alcohol (15 g alcohol and 604 mg polyphenols/can), both lager type. Men drank 660 mL/day (two cans) and women 330 mL/day (one can). The study started with a 4-week adaptation period, followed by a 4-week intervention period, a 4-week washout, and a second 4-week intervention with the other beer. Macrophages stimulated with lipopolysaccharide (LPS) and conditioned with human serum obtained after the intervention with non-alcoholic beer showed a decrease in the expression of IL-1β. This effect was less evident after the consumption of conventional beer, but in this case, a lower protein release of the mature active form of IL-1β was found. TNF-α was also reduced after the intervention with non-alcoholic beer, although this effect was not observed with conventional beer. When overweight and obese participants were compared, it was shown that the decrease in TNF-α was higher in the obese subject group.

Based on these results, the authors concluded that moderate regular intake of non-alcoholic or conventional beer attenuated the inflammasome signaling pathway in human macrophages. However, moderate intake of non-alcoholic beer was associated with a greater anti-inflammatory effect than that produced by conventional beer. In spite of its polyphenol content, the fact that traditional beer has not shown the same ability to modulate inflammatory responses could be due to a possible interaction of polyphenols and the alcoholic fraction.

Very recently, a randomized human trial addressed the effects of moderate consumption of three different beers, with different concentrations of polyphenols, on the composition of the intestinal microbiota [25]. The study involved 20 adults aged 30–60 years, with BMI <40 kg/m^2^. They were classified into healthy subjects and those with metabolic syndrome. After a 2-week washout period, participants were included in a crossover trial to determine the order in which they would receive each of the three interventions: (a) non-alcoholic beer (low polyphenol content: 12.2 mg/100 mL), (b) lager beer (intermediate polyphenol content: 27.83 mg/100 mL; 4.2% alcohol by volume) and (c) dark beer (high polyphenol content: 41.6 mg/100 mL; 4.5% alcohol by volume). Each intervention consisted of consuming a bottle (330 mL) of the corresponding beer once a day for two weeks. The biochemical parameters studied were modified only in the group with metabolic syndrome. Thus, uric acid levels increased significantly, although the values remained within the physiological range. Glycosylated hemoglobin (HbA1c) decreased modestly after the three interventions in this group. On the other hand, HDL-cholesterol levels increased modestly after dark lager ingestion in healthy volunteers. In addition, several changes in the composition of the gut microbiota were observed. Although no differences were found in alpha diversity (diversity of species at the local level) or beta diversity, which reflects the change in the composition of biological communities among groups (basal measurement, dark beer, lager beer and non-alcoholic beer), the consumption of lager beer induced a reduction in the *Verrucomicrobia* phylum, its *Verrucomicrobiaceae* family, and the *Blautia*, *Lachnospira* and *Akkermansia* genus. In the same way, the consumption of non-alcoholic beer decreased the *Verrumicrobia* phylum and the *Ruminococcus* genus. Dark beer was the only beverage that did not lower the levels of *Akkermansia muciniphila*, which has been associated with lower damage induced by glucotoxicity, lipotoxicity, oxidative stress and inflammation. Dark beer could have mitigated the decrease of these bacteria through its rich polyphenol content. In addition, after consuming the different types of beer, significant changes were found in the relative abundance of *Streptococcaceae* and *Streptococcus*. 

In this study, the biochemical pathways affected by beer consumption were also analyzed. Interestingly, a significant decrease in porphyrin metabolism and heme biosynthesis was observed after beer consumption, which was even greater after dark beer consumption. It is important to note that porphyrin metabolism may be increased in obesity, a condition often accompanied by a pro-oxidant and inflammatory state. In addition, the oxidative effects of the heme group can promote dysbiosis and damage to the intestinal epithelium. The authors concluded that the changes induced in gut microbiota by beer consumption seem to be conditioned by its polyphenol content. Some of the observed changes would be related to the antioxidant effects of polyphenols, which could be enhanced by some positive changes in the intestinal microbiota.

## 4. Discussion

Beer is one of the earliest alcoholic beverages in the world. Its composition based on malt, hops and yeast, among others, gives it special chemical properties due to its polyphenol content. However, the presence of alcohol, although low, is a counterpoint. For that reason, the consumption of conventional beer generates controversy regarding its health effects. Consequently, the interest in non-alcoholic beer is growing. It is important to point out that physical methods used in the production of this type of beer can induce the degradation of polyphenols. In fact, some studies [26,27] and the Phenol-Explorer Database [28] have revealed lower phenolic compound amounts in non-alcoholic beer than in conventional beers.

The studies that have analyzed the effects of non-alcoholic beer consumption on the lipid profile focus mainly on the concentrations of total cholesterol, HDL-cholesterol, LDL-cholesterol and triglycerides. In some studies using conventional beer, increased levels of HDL-cholesterol were observed in healthy volunteers (men and women) or in men showing dyslipidemia [18,19,22]. However, in other studies addressing subjects (men and women) who were overweight or obese without other cardiovascular risk factors, no significant effects were found [15,23]. Regarding non-alcoholic beer, only one of the studies included in this review found an increase in HDL-cholesterol [10]. Likely, ethanol contained in conventional beer is involved in the positive effect of this type of beer but, according to the results published, the influence of polyphenols cannot be discarded. 

The classical biomarkers of endothelial dysfunction have been classified into three categories: oxidative markers (e.g., ROS, superoxide anion and nitrotyrosine), inflammatory markers (e.g., soluble adhesion molecules, IL-6, IL- 8, IL-12 and hsCRP) and coagulation pathway markers (e.g., vWF and soluble thrombomodulin) [29]. Regarding oxidative metabolism, non-alcoholic beer intake induces changes that indicate a reduction in oxidative stress [10]. Given that the antioxidant effects of polyphenols have been well described, the decrease in oxidative stress induced by non-alcoholic beer is likely due to its polyphenol content. Concerning the effects of conventional beer, the only study described in the present review that measured parameters related to this process found a tendency towards higher levels of F2-isoprostanesin urine [18]. It is well known that ethanol can increase oxidative stress [30]. Consequently, according to this observation and the data reported by the studies gathered in the present review, non-alcoholic beer seems a better choice than conventional beer from an oxidative status perspective.

The measurement of inflammatory biomarkers has been determined in the initial stages of cardiovascular disease, focusing on its early detection, in order to impact these phases and avoid the development of further complications. Regarding the effects of beer consumption on inflammation-related parameters, controversial results have been reported. While some authors have found no changes in these parameters after non-alcoholic or conventional beer consumption [10,18,23], other authors have observed a significant reduction [12]. Nevertheless, the only study to find an anti-inflammatory effect after beer consumption was in marathon runners, who showed a basal level of inflammation probably higher than the participants in the rest of the studies, due to the well-known exercise-induced inflammation. Concerning the coagulation pathway, both conventional and non-alcoholic beer reduced the expression of the activated fibrinogen receptor, the platelet activation marker CD62 and the formation of monocyte-platelet-aggregate. In addition, non-alcoholic beer yielded a significant inhibitory influence in thrombin generation, whereas beer and ethanol showed procoagulatory effects [14]. Thus, non-alcoholic beer seems to be better than conventional beer from this perspective. 

As far as endothelial function is concerned, in one of the studies using only non-alcoholic beer the consumption of this beverage led to an improvement. This issue was not addressed in the studies that compared non-alcoholic and conventional beer. However, in one of the works in which the effects of non-alcoholic beer, conventional beer and distilled drinks were analyzed, while aortic stiffness was significantly and similarly reduced in all three interventions, endothelial function was only significantly improved after consumption of conventional beer. The authors attributed this effect to the synergy between alcohol and polyphenols. 

Several diabetes-related conditions contribute to increased cardiovascular risk. Among them, insulin resistance and hyperglycemia are the main drivers of atherothrombotic events leading to poor cardiovascular health [31,32]. Taking this into account, it is interesting to analyze the effect of beer intake on parameters related to glycemic control. In this context, some studies have observed an increase in adiponectin levels after conventional beer ingestion, but not following non-alcoholic beer consumption [16,17,19]. Together with the fact that this effect has also been found after gin intake, this suggests that it is induced by the amount of ethanol provided by these beverages. Despite what might be expected from the known positive effects of adiponectin on glycemic control, the change observed in this adipokine did not lead to an increase in insulin sensitivity. Consequently, conventional beer does not seem to be better than non-alcoholic beer with regard to glycemic control.

The present review has some limitations. Unfortunately, only a small number of studies reporting the effects of non-alcoholic beer on cardiovascular health and comparing them with those of conventional beer have been published to date. In addition, there is an array of parameters measured in these studies, which makes it difficult to find a common denominator across studies. Moreover, the sample size of these studies is generally small and participants’ characteristics are also quite variable among studies. Finally, taking into account the duration of the experimental designs, the results might not reflect the potential risks/benefits of longer-term moderate beer consumption.

## 5. Concluding Remarks

The scarce results that describe the effects of non-alcoholic beer consumption suggest that, whereas non-alcoholic beer seems to be superior at preventing oxidative stress in order to preserve endothelial function and inhibit thrombogenic activity, conventional beer is usually able to increase HDL-cholesterol, known as a cardiovascular protective factor. In this regard, it is important to emphasize that HDL-cholesterol can also be increased by other means, such as olive oil intake or physical activity. Thus, the best option for cardiovascular health is probably a combination of non-alcoholic beer intake (instead of conventional beer) together with the inclusion of olive oil in the diet and increased physical activity. Nevertheless, taking into account all the limitations highlighted in the present review with regard to the current knowledge on this issue, further studies to increase the scientific evidence are needed.

## Figures and Tables

**Table 1:** Studies carried out with non-alcoholic beer.

Author	Participants	Intervention	Study and Duration	Measured Parameters	Effects
Martínez- Álvarez et al. (2009) [10]	29 post-menopausal women, aged 58–73 years	Non-alcoholic lager beer	2 intakes of 250 mL/d, each Intervention period: 45 days	Lipid profile: TC HDL-c LDL-c TG) Inflammatory markers: CRP C3, C4 IL-1, IL6 TNFα Parameters of Oxidative metabolism: Oxidized LDL-c TBARS Carbonyl groups Blood antioxidants: Plasma α-tocopherol Erythrocytic GSH	Non-alcoholic beer vs. baseline: ↓ TC ↑ HDL-c Both changes only in hypercholesterolemic participants ↓ Oxidized LDL-c ↓ TBARS ↓ Lipid peroxidation and protein oxidation ↑ α-Tocopherol ↑GSH
López-Jaén et al. (2010) [11]			After 6 months, 400 mg/d of commercial hop Intervention period: 30 days		↓ TC and TG ↓ Oxidized LDL ↓ CRP and IL-6 ↑ α-tocopherol ↓ GSH
Scherr et al. (2012) [12]	277 healthy male marathon runners, aged 20–60 years	Non-alcoholic beer Placebo (without polyphenols)	Intake of 1–1.5 L/d, 3 weeks before, during and 2 weeks after the Munich Marathon race	Inflammation parameters: Serum IL-6 Blood leukocyte counts URTI	↓ IL-6 ↓ Leukocytes ↓ URTI
Macías- Rodríguez et al. (2020) [13]	43 patients with non-alcoholic liver cirrhosis: 21 controls and 22 non-alcoholic beer drinkers (26 women and 17 men), aged 18–70 years	Non-alcoholic beer Water	Intake of 330 mL/d Study duration: 8 weeks	Biochemical parameters: ALT, AST, AP Number of platelets Endothelial dysfunction Hemodynamic variables (heart rate, diastolic, systolic and mean arterial pressure)	↓ AST ↑ Number of platelets ↓ Endothelial dysfunction

ALT: Alanine aminotransferase; AST: Aspartate aminotransferase; AP: Alkaline phosphatase; C3: Complement C3; C4: Complement C4; CRP: C-reactive protein; GSH: reduced glutathione; HDL-c: HDL-cholesterol; IL: interleukin; LDL-c: LDL-cholesterol; TB: Total bilirubin; TBARS: thiobarbiturate acid-reactive substances; TC: total cholesterol; TG: triglycerides; TNF-α: tumor necrosis factor-α; URTI: Upper Respiratory Tract Illness; ↑: significant increase; ↓: significant decrease.

**Table 2:** Studies carried out with both non-alcoholic beer and conventional beer.

Author	Participants	Intervention	Study and Duration	Measured Parameters	Results
Bassus et al. (2004) [14]	12 healthy non-smoker men, aged 19–36 years	Conventional beer Non-alcoholic beer Ethanol	Intake of 3 L of the corresponding beverage in 3 h 4-week rest periods between each intervention	Coagulation-related parameters: PAC-1 CD62 MPA FVIIc ETP Fibrinogen Prothrombin time MPA	Conventional beer vs. baseline: ↑ FVIIc ↓ MPA ↑ ETP Non-alcoholic beer vs. baseline: ↓ PAC-1 ↓ CD62 ↓ MPA ↓ ETP Ethanol vs. baseline: ↑ PAI-1 ↓ MPA ↑ ETP
Imhof et al. (2008) [15]	49 healthy men and women, non-smokers, aged 22–56 years	Beer (5.6% ethanol, polyphenols 169 mg/L) Non-alcoholic beer (polyphenols 171 mg/L) Pure water Amounts equivalent to 30 g/d for men and 20 g/d for women	Intervention period: 3 weeks	Serum lipids: HDL-cholesterol Monocyte migration Inflammatory biomarkers: TNF-α E-selectin ICAM	Conventional beer: No significant effects on HDL-cholesterol, monocyte migration and inflammatory markers Non-alcoholic beer: No significant effects on HDL-cholesterol, monocyte migration and inflammatory markers
Imhof et al. (2009) [16]	72 healthy men and women, non-smokers, aged 22–56 years	Beer (5.6% ethanol, polyphenols 169 mg/L) Non-alcoholic beer (polyphenols 171 mg/L) Pure water Amounts equivalent to 30 g/d for men and 20 g/d for women	Intervention period: 3 weeks	Plasma adiponectin levels	Conventional beer: ↑ Adiponectin Non-alcoholic beer: no significant effect
Beulens et al. (2008a) [17]	20 healthy, non-smoker men, aged 18–25 years	Conventional beer Non-alcoholic beer	3 cans (990 mL)/d Intervention period: 3 weeks with a washout period of 1 week	Serum parameters: Glucose FFA Insulin Glucagon OGTT Adipokines: Adiponectin Ghrelin ASP Leptin Resistin	Conventional beer vs. non-alcoholic beer: ↓ Glucose ↑ Adiponectin ↑ Ghrelin ↓ ASP Insulin: no changes ↑ FFA ↑ Glucagon
Beulens et al. (2008b) [18]	20 healthy, non-smoker men, aged 18–25 years, distributed in normal weight or overweight	Conventional beer Non-alcoholic beer	3 cans (990 mL)/d Intervention period: 3 weeks with a washout period of 1 week	Serum lipids Serum enzymes Serum Lp-PLA2 Blood pressure Serum inflammation parameter: CRP Urinary oxidative stress parameter: F2-isoprostanes	Conventional beer vs. non-alcoholic beer: ↑ HDL-c cholesterol ↓ LDL-c cholesterol ↑ GGT, AST (only in overweight subjects) ↑ F2-isoprostanes (tendency)
Joosten et al. (2011) [19]	24 pre-menopausal healthy, non-smoker women, aged 20–40 years, under treatment with oral contraceptives BMI: 22.2 ± 1.6 kg/m^2^	Conventional beer Non-alcoholic beer	2 cans/d (660 mL)/d Intervention period: 3 weeks with 1-week washout	Serum lipids: HDL-c LDL-c TG FFA Adiponectin Parameters related to insulin resistance Serum enzymes: ALAT, AST, AP, γ-glutamyltrans- peptidase	Conventional beer vs. non-alcoholic beer: ↑ HDL-c ↑ Adiponectin: ↑ γ-glutamyltrans- peptidase No significant effects in serum enzymes
Karatzi et al. (2013) [20]	17 male, healthy volunteers, non-smokers, 28.5 ± 5.2 years of age, BMI = 24.4 ± 2.5 kg/m^2^	Conventional beer (20 g ethanol, 48 mg poly phenols) + water Non-alcoholic beer (48 mg polyphenols) Amounts that provided 30 g/d for men and 20 g/d for women or the same amount of non-alcoholic beer	400 mL/d conventional beer + 400 mL/d water 800 mL/d non-alcoholic beer Intervention periods: 4 weeks	Aortic stiffness Endothelial function Pressure wave reflexes (Aix) Aortic/brachial pressure	Conventional beer: ↓ Aortic stiffness Improvement in endothelial function ↓ Aix ↑ Aortic/brachial pressure Non-alcoholic beer: ↓ Aortic stiffness Improvement in endothelial function ↓ Aix ↑ Aortic/brachial pressure
Chiva- Blanch et al. (2014) [21]	36 men with high cardiovascular risk (DM2, smokers, hypertension, dyslipidemia, overweight or obesity, or a family history of cardiovascular disease), aged 55–75 years	Conventional beer lager type 660 mL/d (30g of alcohol) Non-alcoholic beer lager type	Intervention periods: 4 weeks	Atherosclerosis biomarkers: Progenitor endothelial cells (EPC) Soluble factors	Conventional and non-alcoholic beer: ↑ Number of EPC ↑ Serum soluble stromal cell-derived factor 1
Chiva- Blanch et al. (2015) [22]	36 men with high cardiovascular risk (DM2, smokers, hypertension, dyslipidemia, overweight or obesity, or a family history of cardiovascular disease), aged 55–75 years	Conventional beer lager type 660 mL/d (30 g of alcohol) Non-alcoholic beer lager type 990 mL/d	Intervention periods: 4 weeks	Blood pressure Serum lipid parameters Serum adipokines Coagulation parameters: Fibrinogen, FVIIc, PAI, prothrombin and thromboplastin times Serum and cell adhesion molecules	Conventional beer: ↑ HDL, ApoA-I, ApoA-II ↑ Adiponectin ↓ Serum fibrinogen ↓ Lymphocyte expression of LFA-1 and SLex ↓ Monocyte expression of SLex and CCR2 ↑ IL-1ra ↓ IL-5 Non-alcoholic beer: ↓ Systolic blood pressure ↓ Apo A-I, Apo A-II ↓ Homocysteine ↑ Folic acid ↓ Lymphocyte expression of LFA-1 and SLex ↓ Monocyte expression of SLex and CCR2 ↓ E-Selectin, IL-6r, IL-15 and TNF-β
Padro et al. (2018) [23]	36 subjects (21 men and 15 women), overweight or obese without other cardiovascular risk factors, aged 40–60 years	Conventional lager beer (15 g ethanol and 604 mg polyphenols/can) Non-alcoholic lager beer (0.0 g alcohol and 414 mg polyphenols/can)	2 cans/day (660 mL of beer, i.e., 30 g of alcohol) in men 1 can/day (330 mL of beer, i.e., 15 g of alcohol) in women Intervention period: 4 weeks with 4-week washout period and 4-week adaptation period	Serum lipids: TC HDL-c LDL-c TG Inflammatory parameters: CRP IL-6 TNF-α Vascular parameter: Framingham Risk Score	No significant effects in serum lipids No significant effects in inflammatory parameters No significant effects in vascular parameters
Muñoz- Garcia et al. (2021) [24]	21 men and 15 women aged 40–60 years. Healthy, non-smokers with normal weight or first grade overweight	Conventional beer Non-alcoholic beer	Exposition of cultured macrophages exposed to LPS and to serum obtained after the intervention with conventional beer or non-alcoholic beer	Inflammation parameters: Pro-IL-1β IL-1β TNFα	Conventional beer: ↓ IL-1β protein release Non-alcoholic beer: ↓ IL-1β expression ↓ TNFα

Aix: pressure wave reflexes; ALAT: alanine transaminase; AP: alkaline phosphatase; Apo I and II: apoprotein I and II; ASP: acylation-stimulating protein; AST: Aspartate transaminase; CCR2: C-C chemokine receptor type 2; CD62: cluster of differentiation 62; CRP: C-reactive protein; DM2: diabetes mellitus type 2; EPC: progenitor endothelial cells; ETP: endogenous thrombin potential; FVIIc: factor VII coagulant; FFA: Free fatty acids; GGT: Gamma-glutamyl transferase; ICAM: intercellular adhesion molecule-1; IL-6/1β: interleukin 6/1β; LFA-1: lymphocyte function-associated antigen 1; Lp-PLA2: lipoprotein-associated phospholipase A2; MPA: monocyte platelet aggregates; OGTT: oral glucose tolerance test; PAC-1: first procaspase activating compound; PAI-1: Plasminogen activator inhibitor-1; SDF1: serum soluble stromal cell-derived factor 1; SLex: Sialyl LewisX; TNFα: tumor necrosis factor α; ↑: significant increase; ↓: significant decrease.

## Data Availability

Not applicable.
